# Artificial intelligence and Chinese university teachers’ work performance: a synergistic or adversarial relationship

**DOI:** 10.3389/fpsyg.2025.1698895

**Published:** 2025-11-12

**Authors:** Wenhua Wen, Xinyi Cai

**Affiliations:** 1 Student Affairs Office (Shenzhen Campus), Jinan University, Shenzhen, China; 2 School of Journalism and Communication, Jinan University, Guangzhou, China

**Keywords:** knowledge worker, AI awareness, servant leadership, leader-member exchange, turnover intention, work performance

## Abstract

**Introduction:**

With the widespread adoption of AI by Chinese university teachers in their work processes, an increasing number of complexes work this study aims to examine are being handled by AI. Under these circumstances, as traditional knowledge workers, Chinese university teachers may develop concerns about their career prospects, leading to negative work attitudes and pessimism, which could ultimately affect their work performance.

**Methods:**

Based on the knowledge worker’s perspective of relationship between leader and subordinates and through a self-administered survey, valid questionnaires were collected from 423 Chinese university teachers working in 64 Chinese universities, and partial least squares structural equation modeling (PLS-SEM) was employed for data analysis.

**Results:**

In the result, the study reveals a negative correlation between Chinese university teachers’ AI awareness and LMX, as well as a positive association between servant leadership and LMX. Furthermore, it demonstrates that Chinese university teachers’ LMX is negatively related to turnover intention, which in turn shows a negative relationship with work performance.

**Discussion:**

Against the background of widespread AI adoption in China, this research provides both theoretical implications and practical suggestions for managing, motivating, and inspiring Chinese university teachers to enhance their work performance and thereby improve organizational performance.

## 1 Introduction

With the rapid development of artificial intelligence (AI) technologies in China, university teachers are increasingly integrating AI into their teaching practices, leveraging it to assist in course preparation, assessment, and administrative tasks. Recent studies have demonstrated the growing prevalence of AI in higher education settings, with researchers observing that AI’s application in teaching can significantly enhance efficiency and productivity (Saaida, 2023; Singh and Hiran, 2022). For instance, AI-powered tools can automate grading, recommend personalized learning resources for students, and provide instant feedback, all of which streamline the educational process and allow teachers to focus on more complex aspects of their roles (Gnanaprakasam and Lourdusamy, 2024).

However, despite the potential advantages of AI, some studies suggest that the increasing reliance on AI might result in unintended consequences for employees, including university teachers. The perceived threat of AI replacing human labor has been shown to provoke negative emotions such as anxiety and resentment, which can negatively affect work performance and job satisfaction (Soulami et al., 2024). Research has indicated that workers who perceive AI as a job substitute tend to experience a decline in motivation and engagement, ultimately leading to decreased productivity and well-being (Cramarenco et al., 2023). Hence, given the identified research gap regarding the ambiguous relationship between AI awareness and work performance, this study empirically investigates this dynamic among Chinese university teachers. A positive correlation is interpreted as evidence of a synergistic relationship, while a negative correlation indicates an adversarial one.

Moreover, university teachers are a unique group of knowledge workers, who traditionally have a high degree of autonomy and intellectual engagement in their work (van Dijk et al., 2023). Given their role as knowledge providers, the widespread use of AI in university teaching could lead teachers to fear that their expertise is being undervalued, and that AI might eventually replace them altogether. This perception may contribute to a breakdown in their relationship with their supervisors, fostering negative emotions toward the institution and ultimately diminishing their work performance. According to previous studies on knowledge workers, these employees are characterized by their need for intellectual autonomy, personal development, and meaningful work, all of which could be threatened by AI (Soomro et al., 2024).

Furthermore, the leadership styles that are most effective for knowledge workers, especially in academic settings, remain an underexplored area of research. As university teachers represent a highly specialized group of knowledge workers, it is crucial to investigate the leadership strategies that can enhance their engagement with AI tools while addressing potential concerns related to job security and autonomy. Servant leadership, which emphasizes the well-being and development of employees, has been identified as a promising leadership style for enhancing work performance and emotional well-being in academic environments (Zeng et al., 2022). However, research on the application of servant leadership among Chinese university teachers is still limited, warranting further exploration.

In this study, this study aim to examine the complex relationship between university teachers’ AI awareness and work performance, focusing on how different leadership styles, such as servant leadership, may influence this dynamic. Through a detailed investigation, this study hopes to contribute to the growing body of knowledge on AI in education and provide insights into the leadership strategies that can mitigate the potential adverse effects of AI adoption.

## 2 Literature review

### 2.1 Knowledge workers

The “knowledge worker” concept was proposed by Drucker (1994). He defined knowledge workers as those people who are mastering and applying symbols and concepts and working with knowledge and information.

Knowledge workers usually have a high educational level and professional knowledge and skills. These skills are characterized by high demand, short life cycles, and criticality to the organization, and include symbolic analysis skills (Despres and Hiltrop, 1995), information analysis ability, distribution ability, production capacity, and the ability to use tools or techniques (Dell Acqua et al., 2023).

In the current knowledge-based economy and digital age, the role of knowledge workers has become increasingly crucial. The global economy is increasingly reliant on knowledge workers to meet the needs of both public and private organizations (Bommarito et al., 2023). As such, understanding the productivity, role, and management of knowledge workers is of great significance. Research on knowledge workers encompasses multiple aspects, including their productivity, the role of AI in knowledge work, knowledge management, sociotechnical enablers, and organizational culture. For instance, studies have explored the impact of knowledge management processes on organizational performance with the mediating role of knowledge worker productivity in Chinese higher education (Sahibzada et al., 2022). Additionally, research has investigated the interlinking of networking capabilities, knowledge worker productivity, and digital innovation for sustainable performance in small and medium enterprises (Tariq et al., 2024). These studies collectively contribute to the understanding of the importance of knowledge workers in the contemporary economic and digital landscape.

### 2.2 AI awareness

AI awareness is generally defined as the level of recognition, understanding, and perception that individuals or organizations hold regarding AI technologies, including their potential opportunities, risks, and implications for work (Brougham and Haar, 2018). As noted by He et al. (2023), this concept reflects employees’ recognition of AI as a transformative technological force that can reshape job opportunities, often accompanied by perceived challenges. Typically, such awareness emerges once organizations begin to adopt AI in their operations (Kong et al., 2021). Moreover, AI awareness is associated with new learning experiences, cognitive shifts, and enhanced efficiency, accuracy, and speed—all of which contribute to improved organizational performance (Kumar et al., 2023). When organizations integrate AI into their work processes, employees’ awareness of AI is activated, encouraging deeper engagement in workplace activities and supporting long-term employability (Ding, 2021; Tursunbayeva and Renkema, 2023).

### 2.3 Servant leadership

Servant leadership emphasizes prioritizing the needs of others and underscores the responsibility of organizations to cultivate individuals capable of creating a better future. This perspective resonates strongly with both scholars and practitioners, particularly in light of growing concerns that contemporary business leaders are increasingly self-serving (Canavesi and Minelli, 2022). As Kainde and Mandagi (2023) notes, servant leaders strive to unlock the potential of their followers by fostering their abilities, understanding their needs and aspirations, and supporting the achievement of their goals. Unlike those who pursue leadership primarily out of a desire for authority or material gain, servant leaders adopt a fundamentally different approach—placing service above power. The defining characteristic of servant leadership lies in its reciprocal nature: while followers develop and thrive under servant leaders, leaders themselves increasingly embody the role of servant through this mutual growth process (Kainde and Mandagi, 2023).

### 2.4 Leader-member exchange (LMX)

Rooted in social exchange theory (SET), LMX describes the quality of exchange relationships between leaders and subordinates, highlighting how leaders gradually establish differentiated relationships with various followers within the same group (Dansereau et al., 1975). The LMX framework offers an alternative perspective for examining superior–subordinate interactions by emphasizing that role development naturally results in varied role expectations and exchange patterns between leaders and members.

Most studies on LMX assume that a certain degree of negotiating latitude exists in leader–member interactions. Based on this assumption, LMX quality is generally categorized into two groups: the in-group and the out-group. Members of the in-group enjoy high-quality LMX relationships characterized by trust, support, and both formal and informal rewards (Gerstner and David, 1997). They often receive more frequent feedback, are granted greater autonomy, and may contribute beyond the boundaries of their formal job responsibilities. Conversely, members of the out-group experience low-quality LMX relationships marked by limited trust, minimal support, and fewer incentives. Their interactions with leaders are largely restricted to formal employment requirements, meaning they primarily perform routine tasks and engage with supervisors in a transactional rather than relational manner (Gupta et al., 2025).

### 2.5 Turnover intention

Turnover intention, often regarded as the most immediate antecedent of actual turnover behavior, has been consistently defined as an employee’s conscious and deliberate willfulness to leave the organization (Tett and Meyer, 1993). Mobley (1977) first conceptualized turnover intention as part of a cognitive withdrawal process, where employees evaluate their current job, consider alternatives, and eventually develop an intention to quit. Scholars generally agree that turnover intention is a strong predictor of voluntary turnover and thus serves as a proxy for studying employee attrition (Alkaabi et al., 2024). In higher education settings, particularly among university teachers, turnover intention is viewed as an essential measure of job stability, as it reflects both organizational factors (e.g., leadership style, HR practices) and individual-level determinants (e.g., job satisfaction, work stress, and burnout).

A large-scale survey of teachers in Chinese local undergraduate universities revealed that job stress directly increases turnover intention while also exerting a partial mediating effect through burnout; moreover, teachers’ self-efficacy significantly reduces the impact of job stress on both burnout and turnover intention (Pei et al., 2024). Similarly, in regional higher education institutions, the implementation of high-performance human resource practices (HPHRP)—including talent selection and development, career security, performance appraisal and incentive mechanisms, participatory management, and emotional incentives—significantly reduces teachers’ turnover intention. Among these practices, talent selection and development have the strongest effect, while organizational commitment serves as a mediating factor in this process (Li et al., 2025).

### 2.6 Work performance

Work performance refers to workers’ performance of duties officially accepted as part of their jobs. These actions contribute to the organization’s overall performance both directly (e.g., executing part of a technological process) and indirectly (e.g., supplying required materials or services) (Borman and Motowidlo, 1993).

Work performance is influenced by multiple factors. Job Characteristics Theory posits that intrinsic job characteristics, such as task variety, task significance, autonomy, and feedback, directly affect employee motivation and performance (Violin, 2022). Furthermore, leadership style, job satisfaction, job stress, and organizational support have been found to be closely related to work performance (Yang et al., 2024).

In the higher education sector, teachers’ work performance encompasses not only teaching quality but also research output, academic service, and student mentoring. Studies indicate that teachers’ work performance is influenced by factors such as teaching resources, academic autonomy, peer support, and student feedback (Van Zyl et al., 2024).

## 3 Theoretical framework

**Figure 1:**
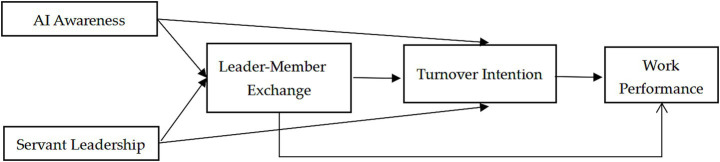
Theoretical framework of the research.

Drawing on SET, this study tests the relationship between Chinese university teachers’ AI awareness, servant leadership, LMX, turnover intention and work performance. Figure 1 illustrates the theoretical framework of this research.

### 3.1 AI awareness and leader-member exchange

AI has become increasingly integrated into higher education, reshaping the way university teachers approach teaching and administrative tasks. As knowledge workers, university teachers rely heavily on their intellectual capital, expertise, and continuous learning to create value for organizations (Drucker, 1994). Compared with other types of employees, knowledge workers are particularly sensitive to changes in the technological environment, as their career prospects and professional identity are closely tied to their ability to apply and update knowledge. Employees’ awareness of AI reflects their perception of how AI adoption may influence their future career opportunities and professional identity (Brougham and Haar, 2018; Teng et al., 2024; Uygun et al., 2024). While AI can enhance efficiency and productivity, it simultaneously generates uncertainty about job security, thereby creating a psychological burden on knowledge workers. Hence, when individuals perceive threats to their valuable resources—such as professional competence or employability—they tend to employ defensive strategies to preserve existing resources or mitigate potential losses (Hobfoll, 2002). In the academic context, the rapid introduction of AI may lead university teachers to interpret its use as a managerial intention to replace them, which heightens stress and triggers defensive attitudes. These reactions may spill over into their interpersonal relationships at work. Specifically, when teachers perceive AI adoption as undermining their role, they may develop mistrust or resentment toward their supervisors, thereby weakening the quality of LMX between them. Hence, this research hypothesizes the negative relationship between AI awareness and LMX.

*H1*: There is a negative relationship between Chinese university teachers’ AI awareness and LMX.

### 3.2 Servant leadership and leader-member exchange

Servant leadership, which emphasizes prioritizing followers’ needs, empowering employees, and building trust-based relationships (Dilley, 2025), is theoretically aligned with the development of high-quality LMX. LMX highlights that leaders form differentiated exchange relationships with subordinates, with high-quality relationships characterized by trust, respect, and mutual obligation (Graen and Uhl-Bien, 1995). Empirical studies demonstrate that servant leaders enhance employees’ perceptions of fairness, trust, and socio-emotional support, which are central to fostering high-quality LMX (Dami et al., 2022). Moreover, servant leadership behaviors, such as humility and individualized care, positively predict employees’ role clarity and psychological safety, which further facilitate stronger leader–member relationships (Van Dierendonck and Nuijten, 2011) Prior research also suggests that servant leadership indirectly promotes organizational outcomes through enhancing LMX quality (Che et al., 2021a; Che et al., 2021b; Nisar Khattak et al., 2024). Hence, this research also hypothesizes the positive relationship between servant leadership and LMX.

*H2*: There is a positive relationship between servant leadership and Chinese university teachers’ LMX.

### 3.3 LMX and turnover intention

A growing body of recent research shows a robust negative association between LMX and turnover intention. Drawing on a social identity lens, a multi-sample study in China demonstrates that high-quality LMX lowers turnover intention via serial mediation through relational and organizational identification, with leader competence strengthening the LMX–relational identification link (Niu et al., 2022). Beyond identity mechanisms, evidence also indicates a double-chained mediation—LMX promotes adjustment to work mode, which reduces exhaustion, thereby decreasing turnover intention (Petrilli et al., 2024). In high-pressure healthcare settings, the “affect” and “professional respect” facets of LMX significantly and negatively predict turnover intention even after accounting for working hours (Saygili et al., 2025). Furthermore, LMX buffers the emotional labor - burnout - turnover intention pathway, mitigating the adverse impact of deep acting on employees’ intentions to quit (Li et al., 2024). Hence, this research also hypothesizes the negative relationship between LMX and turnover intention.

*H3*: There is a negative relationship between Chinese university teachers’ LMX and turnover intention.

### 3.4 Turnover intention and work performance

Recent studies increasingly highlight the detrimental effect of turnover intention on employees’ work performance. Employees who intend to leave often conserve their remaining resources by reducing motivation, engagement, and task-related effort, which results in lower in-role and extra-role performance (Al Muala et al., 2022). A systematic review by Ozkan et al. (2020) further demonstrates that the negative link between turnover intention and performance is robust across contexts, though its strength varies depending on employee type and organizational settings. Longitudinal evidence adds support for a reciprocal mechanism: Lin and Huang (2021) show that performance not only predicts turnover intention but that turnover intention subsequently diminishes performance, suggesting a vicious cycle between the two. In educational contexts, turnover intention has also been found to undermine teachers’ task performance and organizational citizenship behavior (Naim and Ozyilmaz, 2023). Taken together, these findings indicate that turnover intention reduces employees’ willingness and capacity to contribute effectively, thereby threatening organizational performance. Hence, this research also hypothesizes the negative relationship between turnover intention and work performance.

*H4*: There is a negative relationship between Chinese university teachers’ turnover intention and work performance.

### 3.5 AI awareness, LMX and turnover intention

The increasing application of AI in workplaces has significantly impacted employees’ attitudes and behaviors, particularly in terms of AI awareness. Studies show that when employees become aware of AI’s potential impact on their work, they may develop stronger turnover intention, especially when AI is perceived as a threat to job security. However, LMX may play a crucial mediating role in this process.

Research has established that high-quality LMX relationships promote employee organizational commitment and trust, which subsequently reduce turnover intention (Suhendi and Danasasmita, 2024). In the context of AI awareness, employees’ perceptions and understanding of AI influence the quality of their exchange with supervisors, which in turn affects turnover intention. Specifically, higher AI awareness may create a sense of disconnect between employees and their leaders, and if LMX quality is low, employees are more likely to develop turnover intention (Gerstner and David, 1997; He et al., 2023). Based on the theoretical and empirical evidence, this research proposes the following hypotheses:

*H5*: There is a positive relationship between Chinese university teachers’ AI awareness and turnover intention.

*H6*: LMX mediates the relationship between Chinese university teachers’ AI awareness and Turnover intention.

### 3.6 Servant leadership, LMX, and turnover intention

In recent years, servant leadership has gained significant attention as a leadership style that emphasizes meeting employees’ needs, promoting their growth, and building trust. Studies have shown that servant leadership positively influences employees’ attitudes and performance while reducing turnover intention (Liden et al., 2008; Zada et al., 2022). Specifically, servant leadership enhances LMX quality, which in turn fosters organizational commitment and reduces turnover intention (Tursunbayeva and Renkema, 2023).

Building on this foundation, recent research has begun to explore the mediating role of LMX in the relationship between servant leadership and turnover intention. LMX measures the quality of interaction between leaders and members and has been found to increase employees’ sense of belonging, trust, and engagement—all of which effectively reduce turnover intention (Graen and Uhl-Bien, 1995). For instance, Zada et al. (2022) found that high-quality LMX relationships help employees feel more connected to their organizations, leading to lower turnover intention. Therefore, this research proposes the following hypotheses:

*H7*: There is a negative relationship between servant leadership and Chinese university teachers’ turnover intention.

*H8*: LMX mediates the relationship between servant leadership and Chinese university teachers’ Turnover intention.

### 3.7 LMX, turnover intention and work performance

In recent years, LMX theory has gained significant attention, with studies showing that high-quality LMX relationships significantly enhance employees’ work performance (Che et al., 2021a; Che et al., 2021c; Liden et al., 2008). The core of LMX lies in the quality of the interaction between leaders and followers; when this relationship is strong, employees are more likely to demonstrate higher task performance (Che et al., 2021b). At the same time, research also shows that low-quality LMX has a negative relationship with higher turnover intention, which, in turn, negatively impacts work performance (Engström et al., 2025).

As more studies examine the relationship between LMX and work performance, it has become increasingly clear that turnover intention mediates this relationship. Specifically, when employees have poor relationships with their leaders, they tend to develop negative emotions about their work, leading to higher turnover intention. Turnover intention, in turn, reduces employees’ engagement and productivity, ultimately affecting their work performance (Engström et al., 2025). In other words, turnover intention may weaken employees’ motivation and performance, particularly in high-pressure environments. Hence, this research proposes the following hypotheses:

*H9*: There is a positive relationship between Chinese university teachers’ LMX and work performance.

*H10*: Turnover intention mediates the relationship between Chinese university teachers’ LMX and work performance.

## 4 Research method

### 4.1 Sampling

According to Sekaran and Bougie (2003), a sample must comprise an adequate number of suitable individuals from the target population to ensure accurate estimation of population parameters. The data collection for this study spanned a twenty-week period. Using a simple random sampling method, initial contact was made with 587 eligible university teachers from 93 higher education institutions across mainland China through organizational recruitment channels such as university administrative offices and departmental heads. All invited participants were required to complete an online questionnaire survey. After implementing a data screening procedure to exclude invalid responses (including incomplete questionnaires and straight-line responses), 423 valid questionnaires from 64 universities were ultimately retained for analysis, yielding a valid response rate of 72%.

Through organizational recruitment channels, this study initially invited 587 qualified university teachers from 93 Chinese higher education institutions to participate in the investigation. Ultimately, 423 respondents from 64 universities agreed to participate and completed the questionnaire, yielding a response rate of 72%.

### 4.2 Data collection method

Data were collected using a self-administered questionnaire that was distributed to Chinese university teachers in China. Measurement items in the questionnaire were adopted from previous researches. All measurement scales were adopted from well-established instruments whose reliability and validity have been confirmed in prior research. Consequently, no items were modified for this study. As this study was conducted in China, the original items were translated into Chinese. The translated items were then verified by linguistic experts to ensure translation accuracy.

AI awareness was adopted from Uygun et al. (2024). These 10 questions were asked on a five-point Likert scale anchoring from (1) strongly disagree to (5) strongly agree. The sample items are: “I am clear about the specific applications of AI in my work environment. I believe that AI can help me improve my work efficiency. I understand the main functionalities and limitations of the AI tools used by my company. I am concerned that AI could replace my job position in the future.”

Servant leadership was adopted from Sendjaya et al. (2019). These 6 questions were asked on a five-point Likert scale anchoring from (1) strongly disagree to (5) strongly agree. The sample items are: “My supervisor uses power in service to others, not for his or her ambition. My supervisor Gives me the right to question his or her actions and decisions. My supervisor helps me to generate a sense of meaning out of everyday life at work.”

LMX was adopted from Graen and Uhl-Bien (1995). These 7 questions were asked on a five-point Likert scale anchoring from (1) strongly disagree to (5) strongly agree. The sample items are: “I usually know how satisfied my supervisor is with what I do. My supervisor recognizes my potential. I would defend and justify my supervisor’s decision if he/she were not present to do so.”

Turnover intention was adopted from Bothma and Roodt (2013). These 6 questions were asked on a five-point Likert scale anchoring from (1) strongly disagree to (5) strongly agree. The sample items are: “I foresee that I will leave this company within the next year. If I had my way, I would be working for a different company tomorrow morning. I do not plan to be with this company indefinitely.”

Work performance was adopted from Koopmans et al. (2014). These 7 questions were asked on a five-point Likert scale anchoring from (1) strongly disagree to (5) strongly agree. The sample items are: “I managed to plan my work so that it was done on time. I kept in mind the results that I had to achieve in my work. I was able to perform my work well with minimal time and effort.”

### 4.3 Data analysis techniques

The data collected through self-administered questionnaires were analyzed using partial least squares structural equation modeling (PLS-SEM). This analytical technique allows for the concurrent testing of multiple interrelated hypotheses by capturing and modeling the complex associations among latent constructs (Anderson and Gerbing, 1988).

## 5 Result

### 5.1 Demographic profile

The demographic profile of the participants basically shows the general background of respondents. Table 1 showed the details of the demographic characteristics of the respondents in this study. As shown in Table 1, a total of 423 valid responses were collected. The sample was relatively balanced in gender, with 48.2% male (*n* = 204) and 51.8% female (*n* = 219). Most respondents were between 31 and 40 years old (56.7%, *n* = 240), while 18.2% (*n* = 77) were aged 30 or below, 18.0% (*n* = 76) were 41–50, and 7.1% (*n* = 30) were 50 or above. In terms of education, the majority held doctoral degrees (79.2%, *n* = 335), followed by master’s (19.9%, *n* = 84) and bachelor’s (0.9%, *n* = 4). Regarding academic position, 70.0% (*n* = 296) were associate professors, 20.1% (*n* = 85) professors, and 9.9% (*n* = 42) lecturers. Geographically, respondents were distributed across northern (39.2%), southern (31.9%), central (14.7%), and eastern (14.2%) China.

**Table 1:** Demographic characteristics of the respondents.

Demographic variables	Category	Frequency	Percent (%)
Gender	Male	204	48.2
	Female	219	51.8
Age	≤30	77	18.2
	31–40	240	56.7
	41–50	76	18
	≥50	30	7.1
Education	Bachelor	4	0.9
	Master	84	19.9
	Doctor of Philosophy	335	79.2
Position	Lecturer	42	9.9
	Associate Professor	296	70.0
	Professor	85	20.1
School area	North area (Northeast China, Northwest China and North China)	166	39.2
	East area (East China)	60	14.2
	South area (South China and Southwest China)	135	31.9
	Central area (Central China)	6	14.7

### 5.2 Composite reliability and convergent validity

The evaluation of the measurement model represents the initial stage in PLS-SEM. As suggested by Hair et al. (2014), a composite reliability value exceeding 0.7 is considered acceptable. As presented in Table 2, the composite reliability values for AI awareness, servant leadership, LMX, turnover intention, and task performance all surpass the 0.7 threshold, thereby satisfying the criterion for establishing convergent validity.

**Table 2:** The result of composite reliability.

Model construct	Items	Composite reliability
AI awareness	10	0.927
Servant leadership	6	0.902
LMX	7	0.905
Turnover intention	6	0.878
Work performance	7	0.879

Second, the convergent validity of the model must be assessed. According to Hair et al. (2014), convergent validity is established when factor loadings exceed 0.50 and the average variance extracted (AVE) surpasses 0.50. As shown in Table 3, the factor loadings of AI awareness, servant leadership, LMX, turnover intention, and task performance are all above 0.50, and the AVE values of these constructs likewise exceed the 0.50 threshold. These results confirm that all constructs demonstrate satisfactory convergent validity.

**Table 3:** The result of convergent validity.

Model construct	Measurement items	Loading	AVE
AI awareness	AI1	0.838	0.605
	AI2	0.768	
	AI3	0.769	
	AI4	0.749	
	AI5	0.742	
	AI6	0.731	
	AI7	0.779	
	AI8	0.835	
	AI9	0.782	
	AI10	0.779	
Servant leadership	SL1	0.802	0.671
	SL2	0.820	
	SL3	0.798	
	SL4	0.866	
	SL5	0.784	
	SL6	0.843	
LMX	LMX1	0.805	0.636
	LMX2	0.796	
	LMX3	0.798	
	LMX4	0.783	
	LMX5	0.789	
	LMX6	0.791	
	LMX7	0.820	
Turnover intention	TI1	0.791	0.622
	TI2	0.782	
	TI3	0.786	
	TI4	0.862	
	TI5	0.730	
	TI6	0.777	
Work performance	WP1	0.757	0.580
	WP2	0.771	
	WP3	0.765	
	WP4	0.743	
	WP5	0.839	
	WP6	0.720	
	WP7	0.732	

Where the AI, AI awareness; SL, servant leadership; LMX, leader-member exchange; TI, turnover intention; WP, work performance.

### 5.3 Discriminant validity

Discriminant validity is an essential component of construct validity. The HTMT rate of factors to measure discriminant validity between similar and different indicators. In order to fulfill discriminant validity, HTMT values must be lower than 0.85 (Henseler et al., 2015). As presented in Table 4, all values are lower than 0.85, indicating that the discriminant validity of all five variables is both acceptable and well established.

**Table 4:** Discriminant validity of constructs (HTMT).

Constructs	AI awareness	LMX	Servant leadership	Turnover intention	Work performance
AI awareness	**-**	**-**	**-**	**-**	**-**
LMX	0.664	**-**	**-**	**-**	**-**
Servant leadership	0.404	0.637	-	**-**	**-**
Turnover intention	0.615	0.740	0.605	**-**	**-**
Work performance	0.595	0.716	0.617	0.743	**-**

### 5.4 Analysis of the structural model

**Figure 2:**
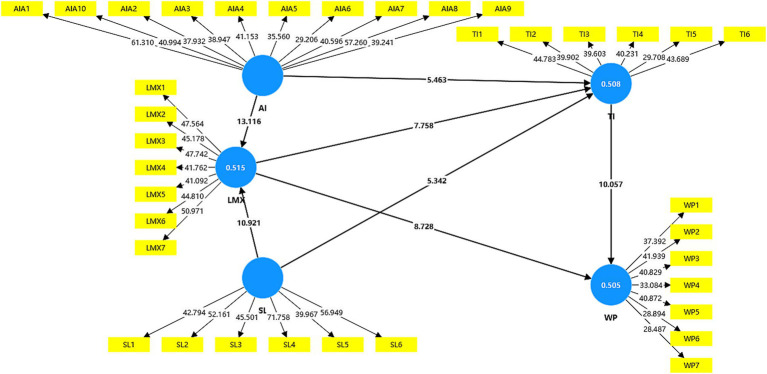
The structural model for individual latent variable.

R^2^ values are commonly used to assess the explanatory power of each construct within the structural model. According to Hair et al. (2014), R^2^ values ranging between 0 and 1 are considered acceptable. As illustrated in Figure 2, the *R*^2^ values of LMX, turnover intention, and work performance as endogenous variables are 51.5, 50.8, and 50.5% respectively, indicating a substantial level of explanatory power.

Furthermore, communality and redundancy values can be examined as supplementary indicators of structural model specification. A structural model is considered more robust when communality exceeds 0.40 and redundancy remains exceeds 0.26. The specification values of all five variables are presented in Table 5.

**Table 5:** Structural model specification.

Constructs	*R* ^2^	Communality	Redundancy
AI awareness	Predictor	0.506	Predictor
Servant leadership	Predictor	0.537	Predictor
LMX	0.516***	0.511	0.323
Turnover intention	0.435***	0.470	0.312
Work performance	0.505***	0.438	0.288

Significant level *R*^2^: > 0.32 (substantial)***, > 0.15 (moderate)**, > 0.02 (weak)*.

As shown in Table 5, all constructs satisfy the criteria for communality. With respect to redundancy, the values for LMX, turnover intention, and work performance also meet the required thresholds. Accordingly, all constructs fulfill the conditions necessary for verifying the structural model.

### 5.5 Hypothesis testing

**Figure 3:**
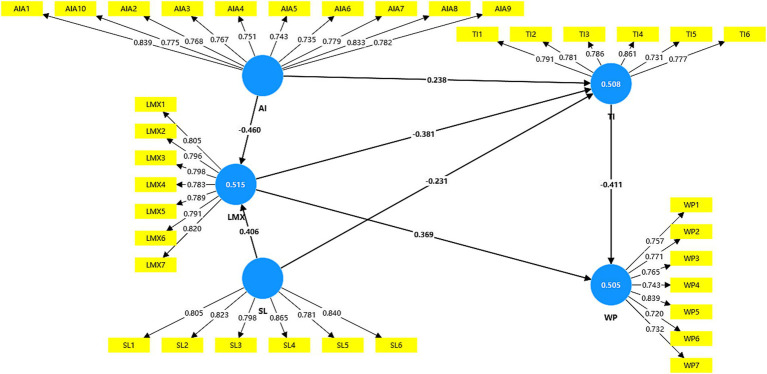
Hypothesis testing of the model.

The hypotheses were tested through bootstrapping, a procedure that involves repeated random sampling with replacement from the original dataset to generate bootstrap samples. The resulting standard errors are then employed to assess the significance of each hypothesis. Figure 3 illustrates the application of the PLS bootstrapping method to the model.

Table 6 reports the results of the hypothesized structural relationships among AI awareness, servant leadership, LMX, turnover intention, and work performance. At the 1% significance level of a two-tailed test, a *t*-value of 2.58 or higher is required. The findings indicate that AI awareness is significantly associated with LMX (*t* = 13.116) and turnover intention (*t* = 5.463). Similarly, servant leadership shows significant associations with LMX (*t* = 10.921) and turnover intention (*t* = 5.342). In addition, LMX is significantly related to turnover intention (*t* = 7.758) and work performance (*t* = 8.728). Finally, turnover intention is significantly associated with work performance (*t* = 10.057). Collectively, these results provide empirical support for hypotheses H1, H2, H3, H4, H5, H7 and H10.

**Table 6:** Hypothesis testing.

Hypotheses	Relationship	Standard deviation	T Statistics	*p*-value	Supported
H1	AI – LMX	0.035	13.116	0.000***	Yes
H2	SL – LMX	0.037	10.921	0.000***	Yes
H3	LMX – TI	0.049	7.758	0.000***	Yes
H4	TI – WP	0.041	10.057	0.000***	Yes
H5	AI – TI	0.044	5.463	0.000***	Yes
H7	SL – TI	0.043	5.342	0.000***	Yes
H9	LMX – WP	0.042	8.728	0.000***	Yes

***Significant at *p* < 0.01 at a two-tailed T statistics value of 2.58. Where the AI, AI awareness; SL, servant leadership; LMX, leader-member exchange; TI, turnover intention; WP, work performance.

To examine the mediating effects of LMX and turnover intention on the relationships among AI awareness, servant leadership, and work performance, this study calculated the variation accounted for (VAF), which indicates the proportion of the direct effect absorbed by the mediating variable. A VAF value below 0.20 suggests no mediation, values between 0.20 and 0.80 indicate partial mediation, and values above 0.80 reflect full mediation. As presented in Table 7, LMX partial mediates the relationships between AI awareness and turnover intention, thereby supporting H6 (VAF = 0.424). Besides, LMX partial mediates the relationships between servant leadership and turnover intention, thereby supporting H8 (VAF = 0.402). In addition, turnover intention partial mediates the relationship between LMX and work performance (VAF = 0.299), providing support for H10.

**Table 7:** Testing the mediation effect.

Hypotheses	Relationship	T statistics	*p*-value	VAF	Supported
H6	AI - LMX - TI	6.608	0.000*	0.424	Yes
H8	SL - LMX - TI	6.134	0.000*	0.402	Yes
H10	LMX - TI - WP	6.580	0.000*	0.299	Yes

*Significant: *p* < 0.10; **Significant at *p* < 0.05; ***Significant at *p* < 0.01. AI = AI awareness. SL = servant leadership. LMX = leader-member exchange. TI = turnover intention. WP= work performance.

## 6 Discussion

Against the backdrop of the widespread adoption of AI in Chinese universities, this study investigates the relationships among AI awareness, servant leadership, LMX, turnover intention, and work performance by focusing on university teachers as a representative group of knowledge workers. Using survey data, the study explores these relationships from both theoretical and empirical perspectives. In the end, the results of this study indicate an negative relationship between Chinese university teachers’ AI awareness and their work performance, suggesting an adversarial relationship between the two constructs.

While the relationship between AI awareness and LMX has not been empirically examined, some studies have explored its connections with various negative outcome variables. For example, Teng et al. (2024) investigated that AI awareness is negatively related to work-related rumination and emotional exhaustion among hotel employees. In the same time, this research reveal that university teachers’ AI awareness exerts a negative effect on LMX. In the context of the rapid development and diffusion of AI technologies, teachers’ perceptions and attitudes toward AI significantly influence their work-related emotions and interpersonal exchanges. When teachers perceive AI as a potential threat, they may attribute the introduction of AI to organizational and leadership intentions to replace their roles, leading to negative attitudes toward their leaders. Such perceptions undermine the quality of leader–subordinate relationships, increase turnover intention, and ultimately reduce work performance. Therefore, it is recommended that university leaders engage in proactive communication with faculty members when introducing AI-related policies, alleviating concerns about job substitution, and reinforcing the role of AI as a supportive rather than replacement tool, thereby maintaining trust and relational stability.

The results further indicate that servant leadership has a positive impact on LMX, consistent with the findings of Nisar Khattak et al. (2024) and El-Bayaa (2025). Particularly, while Nisar Khattak et al. (2024) demonstrated a positive correlation between servant leadership and LMX among service employees in the U. S. hospitality industry, this study confirms that such relationship remains valid within the context of Chinese knowledge workers. Hence, by emphasizing humanistic care and personal development, servant leaders act as both mentors and service providers, fostering harmonious leader–subordinate relationships. For knowledge workers, such relationships strengthen affective bonds and a sense of responsibility, encouraging them to reciprocate through greater work effort, thus reducing turnover intention and enhancing work performance. This suggests that leaders in Chinese universities should adopt servant leadership practices rather than relying on authoritarian command, thereby effectively motivating and guiding faculty members.

Moreover, the study confirms the positive relationship between LMX and work performance, echoing recent empirical findings (Ahmed et al., 2024; Henderson and Jeong, 2024; Ionescu and Iliescu, 2021). For example, Ahmed et al. (2024) collected data from 295 employees and their supervisors working in various public sector organizations of Pakistan and proved that high LMX can improve their task performance. High-quality LMX enhances employees’ sense of comfort and organizational belonging, which in turn stimulates greater task engagement and performance outcomes.

Further analysis shows that turnover intention mediates the relationship between LMX and work performance. The negative correlation between LMX and turnover intention aligns with the findings of Petrilli et al. (2024), whose research was conducted in Catholic University, Paris. This study also confirms that turnover intention is negatively correlated with task performance, thereby supporting the established findings of Kaur et al. (2024). Kaur et al. (2024) investigated that academic teachers’ turnover intention have a negative influence on their work performance in India. Hence, based on the previous studies, the finding of this research pointed out that strong leader–member relationships reduce employees’ intention to leave, which subsequently promotes higher work performance. Taken together, these findings underscore the importance for university leaders to foster supportive and trust-based leader–member interactions, thereby mitigating faculty turnover risks and sustaining organizational performance.

Despite the extensive application of AI in China, the profound influence of traditional culture may still foster resistance to AI technologies among knowledge workers, particularly among university faculty. Therefore, this study also recommends that higher education institutions engage in thorough communication with teachers when developing relevant policies and curricula, aiming to mitigate their negative perceptions toward AI.

### 6.1 Contribution

The findings of this study offer important implications for management practices in higher education institutions. As typical knowledge workers, university teachers seek to achieve self-worth through their professional knowledge while gaining respect and recognition. They also demonstrate distinct independence and autonomy—being unwilling to submit to rigid constraints nor blindly advocate authority. Against the backdrop of AI’s comprehensive integration into education, these characteristics may lead to adaptive anxiety among university teachers toward technological changes, consequently affecting their work performance. Therefore, there is an urgent need to reconstruct the interaction between administrators and teachers through innovative leadership models (servant leadership).

This study empirically investigates the mechanistic relationships among AI awareness, servant leadership, LMX, turnover intention, and work performance. The results not only theoretically reveal behavioral patterns of knowledge workers as core drivers of university development, but also provide empirical evidence and practical approaches for Chinese universities to effectively motivate, manage, and empower university teachers.

### 6.2 Limitations

This study has a number of limitations. First of all, in this study, the sample was exclusively drawn from Chinese higher education institutions, a sampling characteristic that may constrain the generalizability of the findings across diverse sectors. Subsequent research could expand the sample scope to include multiple industries such as the service sector, internet industry, and manufacturing sector to derive more universally applicable conclusions.

Second, as the empirical investigation was exclusively conducted within the Chinese context, the applicability of the findings across diverse socioeconomic backgrounds and cultural environments requires further verification. Future research should incorporate cross-national comparative analyses and extend the participant pool to include non-teaching professional staff within higher education institutions (e.g., administrative personnel, research assistants) to enhance the generalizability and comprehensiveness of the conclusions.

Furthermore, although the data were collected through a convenience sampling method from multiple Chinese higher education institutions, the sample size remains relatively limited. Consequently, the findings are constrained to the current research sample and cannot be generalized to the broader population of Chinese universities. Future studies should incorporate more diverse occupational groups and multicultural contexts to validate and extend the generalizability of the research outcomes.

Despite the aforementioned limitations, the empirical findings of this study retain significant value: First, the research validates the critical role of LMX within the context of Chinese higher education institutions. Second, it reveals innovative leadership (servant leadership) paradigms particularly suitable for managing knowledge workers in academic settings, specifically university teachers. Finally, the study elucidates the mechanistic pathway through which AI awareness influences knowledge workers’ (i.e., university teachers’) LMX with supervisors, subsequently affecting their turnover intention and work performance, and ultimately contributing to long-term organizational effectiveness.

## 7 Conclusion

This empirical study systematically investigates the mechanistic relationships among AI awareness, servant leadership, LMX, turnover intention, and work performance in the context of Chinese higher education institutions. Based on data collected from 423 university teachers across 64 universities, the findings reveal that: (1) AI awareness demonstrates a significant negative correlation with LMX; (2) Servant leadership shows a significant positive correlation with LMX; (3) LMX is negatively correlated with turnover intention while positively correlated with work performance; (4) Turnover intention and work performance exhibit a significant negative correlation. Further analysis identifies LMX and turnover intention as sequential mediators in the relationships among AI awareness, servant leadership, and work performance.

The study contributes to SET and LMX literature by demonstrating the importance of establishing high-quality supervisor-subordinate relationships and validating the effectiveness of servant leadership in academic management contexts. Practical implications suggest that university administrators should: first, establish transparent communication mechanisms to facilitate rational understanding of AI’s educational value and alleviate technological anxiety; second, adopt servant leadership patterns to enhance LMX quality, thereby reducing turnover intention and ultimately achieving sustainable improvement in teaching and research performance.

## Data Availability

The original contributions presented in the study are included in the article/supplementary material, further inquiries can be directed to the corresponding author.
