# Correction: Artificial intelligence and Chinese university teachers' work performance: a synergistic or adversarial relationship

**DOI:** 10.3389/fpsyg.2025.1749983

**Published:** 2025-12-01

**Authors:** Wenhua Wen, Xinyi Cai

**Affiliations:** 1 Student Affairs Office (Shenzhen Campus), Jinan University, Shenzhen, China; 2 School of Journalism and Communication, Jinan University, Guangzhou, China

**Keywords:** knowledge worker, AI awareness, servant leadership, leader-member exchange, turnover intention, work performance

The order of authors in the author list of the published paper was erroneously given as:

Xinyi Cai^1*^ and Wenhua Wen^2^

The correct author list reads:

Wenhua Wen^1^ and Xinyi Cai^2*^

The original version of this article has been updated.

